# Breeding progress, environmental variation and correlation of winter wheat yield and quality traits in German official variety trials and on-farm during 1983–2014

**DOI:** 10.1007/s00122-016-2810-3

**Published:** 2016-10-27

**Authors:** Friedrich Laidig, Hans-Peter Piepho, Dirk Rentel, Thomas Drobek, Uwe Meyer, Alexandra Huesken

**Affiliations:** 1 Bundessortenamt, Osterfelddamm 80, 30627 Hannover, Germany; 2 Biostatistics Unit, Institute of Crop Science, University of Hohenheim, Fruwirthstrasse 23, 70599 Stuttgart, Germany; 3 Department of Safety and Quality of Cereals, Federal Institute of Food and Nutrition, Schuetzenberg 12, 32756 Detmold, Germany

## Abstract

**Key message:**

**Over the last 32** **years, a large gain in grain yield (24** **%) was achieved in official German variety trials, and despite considerable loss in protein concentration (−7.9** **%), winter wheat baking quality was partially improved over the last 32** **years. On-farm gain in grain yield (32** **%) exceeded gain in trials, but at yield level about 25** **dt** **ha** ^**−1**^ **lower. Breeding progress was very successfully transferred into both progress in grain yield and on-farm baking quality.**

**Abstract:**

Long-term gains in grain yield and baking quality of 316 winter wheat varieties from German official trials were evaluated. We dissected progress into a genetic and a non-genetic part to quantify the contribution of genetic improvement. We further investigated the influence of genotype and environment on total variation by estimating variance components. We also estimated genetic and phenotypic correlation between quality traits. For trial data, we found a large gain in grain yield (24%), but a strong decline in protein concentration (−8.0%) and loaf volume (−8.5%) relative to 1983. Improvement of baking quality could be achieved for falling number (5.8%), sedimentation value (7.9%), hardness (13.4%), water absorption (1.2%) and milling yield (2.4%). Grain yield, falling number and protein concentration were highly influenced by environment, whereas for sedimentation value, hardness, water absorption and loaf volume genotypes accounted for more than 60% of total variation. Strong to very strong relations exist among protein concentration, sedimentation value, and loaf volume. On-farm yields were obtained from national statistics, and grain quality data from samples collected by national harvest survey. These on-farm data were compared with trial results. On-farm gain in grain yield was 31.6%, but at a mean level about 25 dt ha^−1^ lower. Improvement of on-farm quality exceeded trial results considerably. A shift to varieties with improved baking quality can be considered as the main reason for this remarkable improvement of on-farm baking quality.

**Electronic supplementary material:**

The online version of this article (doi:10.1007/s00122-016-2810-3) contains supplementary material, which is available to authorized users.

## Introduction

Breeding for improved baking quality of winter wheat was very successful in Germany after World War II. The introduction of shorter varieties (genotypes) allowed higher levels of nitrogen application as well as late top dressing, and together with the release of varieties with better protein quality it was possible to produce winter wheat with acceptable baking quality. Since returning to self-sufficiency after World War II, Germany still had to import about 2 million tons of high quality baking wheat from Canada every year until the 1970s. In the course of the1970s, however, winter wheat production in Germany was able to cover the domestic demand of wheat with sufficient baking quality (Porsche 2008). Today, self-sufficiency has reached about 130% (StatJ 2015).

Winter wheat is the most important crop in Germany with a growing area of about 3.2 million ha (Besondere Ernte- und Qualitaetsermittlung (BEE) 2014), which corresponds to 27% of arable land (StatJ 2015). The total grain production of winter wheat reached 27.4 million tons in 2014 (Besondere Ernte- und Qualitaetsermittlung (BEE) 2014). About 33% of national wheat consumption is used for milling and bread making and 51% for animal feed (StatJ 2015).

The German wheat classification system grades varieties according to their baking quality as part of the registration process. E-grade (elite) wheats have the highest quality, followed by A-grade (quality), B-grade (bread making) and C-grade (not useable for baking) wheats, the latter have the lowest quality. Allocation to a certain quality group is dependent on particular minimum requirements with respect to individual quality traits (Bundessortenamt 2015, p. 126), i.e. loaf volume, falling number, crude protein concentration, sedimentation value, water absorption and milling yield (T550), and on the comparison with a defined reference variety. Finally, the relation or difference of a variety’s quality trait to a defined reference variety is relevant.

**Fig. 1:**
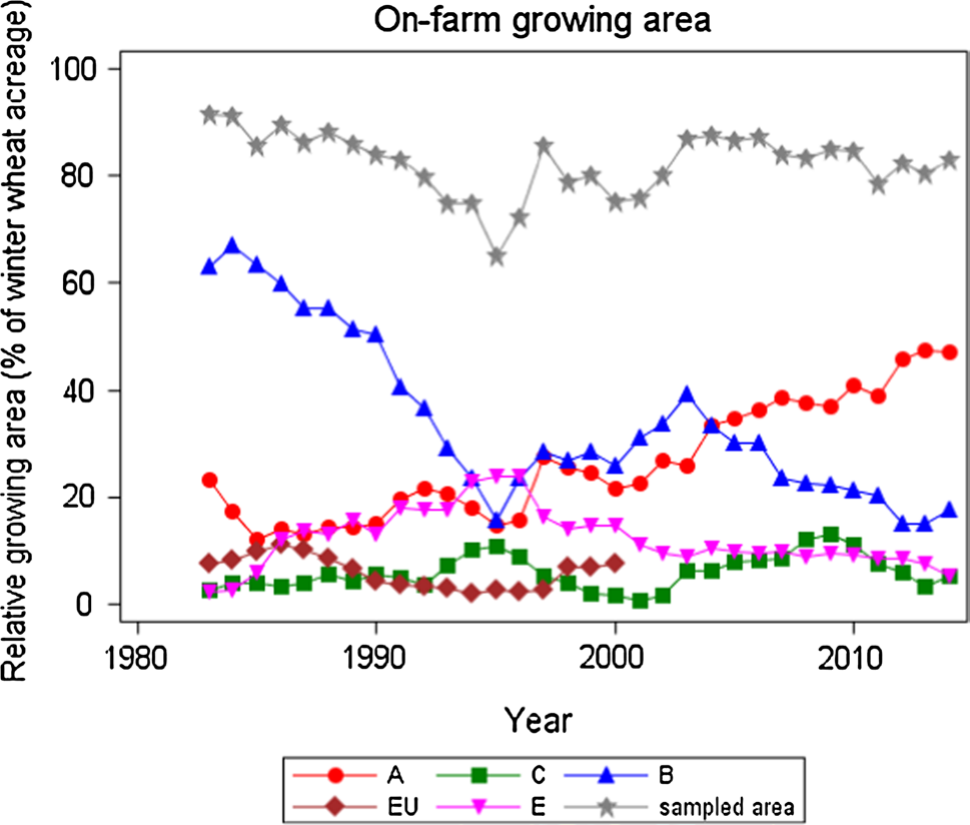
National growing area of winter wheat quality groups as percentage of total winter wheat acreage. Sampled area: total of sampled area from which varieties were reported; *A*, *B*, *C* and *E* varieties with quality group *A, B, C, E*; *EU* varieties from other *EU*-countries, not quality graded

Due to their contribution to end-use quality, grain yield and grain protein concentration are the most important traits determining the economic value of a bread wheat crop (Oury and Godin 2007). The market price for winter wheat varieties with baking quality depends on the protein concentration and the quality grading. For fodder quality (C-grade), the average producer price (2010–2014) at the end of August was 17.23 € per dt (Erntebericht 2014). Farmers receive an average extra payment of 1 € per dt for B compared to C, 1 € for A compared to B and of 2.50 € for E compared to A-grade wheat. Due to these price incentives, a major shift in quality grades grown on-farm occurred (Fig. 1). From 1983 to mid-1990s, the percentage of B-grade varieties decreased drastically to less than 20%, whereas the growing area for A-grade and E-grade varieties increased. After the mid-1990s, the percentage of A-grade continually increased. From Fig. 1, it can be seen that about 50% of the wheat growing area in Germany today is covered by A-grade varieties and about 7% by E-grade. In VCU trials, no such shift to higher quality grades occurred (Electronic Appendix Fig. S1).

Baking quality of winter wheat is mostly determined by protein concentration and quality. The major endosperm protein, gluten, is responsible for bread making quality. The genetically determined composition of gluten is the main determinant of genotypic differences in baking quality (Payne et al. 1987). Seling (2010) points out that the protein quality is genotype specific but can be influenced by some non-genetic factors, i.e. an extreme lack of sulphur. Tannhaeuser et al. (2014) conclude that all constituents of wheat flour, not only proteins, affect baking performance. But it is agreed that the most important contribution to baking performance has to be ascribed to gluten.

Unfortunately, a well-known strong negative relation exists between grain yield and protein concentration. Many studies focused on this negative relation and investigated its genetic basis (e.g. Simmonds 1995; Hartl et al. 2011; Brancourt-Hulmel et al. 2003; Oury and Godin 2007; Oberforster and Werteker 2011; Souza et al. 2012; Sherman et al. 2014; Rozbicki et al. 2015). This relation is essential to breeding progress in grain yield on the one hand and baking quality on the other hand.

Results reported in the literature generally agree that considerable gain in grain yield was achieved, but are inconsistent as to whether or not significant progress in baking quality has been made during the last three to four decades (Cox et al. 1989; Uzik et al. 2009; Hartl et al. 2011).

Grain yield and wheat quality are subject to a complex interaction between genotype and many environmental factors. Important factors are total nitrogen supply, rainfall, temperatures during ripening and soil fertility. Results from numerous studies on the influence of genotype and environment on winter wheat baking quality are reported in the literature (Baenziger et al. 1985; Lukow and McVetty 1991; Peterson et al. 1992, 1998; Graybosch et al. 1996; Finlay et al. 2007; Hristov et al. 2010; Dencic et al. 2011; Vazquez et al. 2012; Kaya and Akcura 2014; Bilgin et al. 2015; Rozbicki et al. 2015). Williams et al. (2008) reviewed 100 publications reporting on the influence of genotype and environment on wheat quality which showed that variation of the relative contribution of genotype, environment, and genotype by environment interaction was highly dependent on the genotypes and environments sampled. Therefore, results from different studies may be quite divergent. The review found that in North America and Europe, traits associated with protein concentration were more influenced by environment and genotype by environment interaction than those associated with protein quality, dough rheology and starch characteristics, where genotype effects were more important.

In this paper, we study trends in yield and baking quality of winter wheat varieties tested and released during the last 32 years in Germany. We first describe the datasets analysed and methods applied. Besides grain yield, ten important quality traits for winter wheat are considered. We quantify the progress in terms of gains or declines in grain yield and quality traits, pay attention to dissecting genetic and non-genetic sources of trend and compare results of trials assessing the value for cultivation and use (VCU trials) with on-farm results obtained from national harvest survey. Genotypic and environmental variation of grain yield and quality traits will be quantified. We further analyse the relationship between traits studied in terms of phenotypic and genotypic correlations.

## Materials and methods

### Data sets

#### VCU trial data

Newly bred candidate varieties must be evaluated for their value of cultivation and use (VCU) before they can be registered on the National List and released for commercial production. Important performance traits are yield, quality traits and disease resistance. Each year in Germany, more than 100 winter wheat (*Triticum aestivum* L.) candidates enter VCU trials to potentially become registered. Only about 15–20% of the candidate varieties are finally released. After registration, varieties are tested usually for two further years in regional trials run by federal states before they are recommended for on-farm use.

The statutory VCU trial period for winter wheat candidate varieties lasts three years. Varieties were grown at up to 30 locations with 2–3 replications. The average harvested plot size was 11.6 m^2^. Trials were about equally distributed across an individual crop’s typical growing region in Germany. Two to three intensities of fertilizer and fungicide treatments were applied. Grain yield and quality were assessed from the intensity comprising best local agronomic practice in fertilizer, fungicide and other agrochemical treatment.

Bulked samples for laboratory tests of quality traits were taken from eight locations every year. Grain yield data were assessed from the same locations and the same intensity as the samples for laboratory analysis were drawn from. Before 1990, only data from West German locations were available for our study. Varieties which were withdrawn or rejected were eliminated from the dataset. We analysed only those varieties which were registered with approved value for cultivation and use. Four varieties with special properties for organic farming have not been included in the data set. At least three standard varieties running in trials for several years were grown together with candidate varieties in each single trial. Well-established varieties were chosen as standards representing the actual state of breeding progress in agronomic and quality traits.

The VCU data set used in this study contained 316 released varieties, including 40 standard varieties. Besides grain yield, ten quality traits were studied (Table 1). The number of observations per trait was between 10,231 and 11,930. The oldest standard variety was first tested in 1963, i.e. that the time of a varieties’ first year in trial spanned a period from 1963 to 2012; this covers 50 years of breeding.

**Table 1:** Investigated traits

Source	Trait	Abbreviation	Unit	Test type	Description
VCU	Grain yield at 86% dry matter	GRAIN_Y	dt ha^−1^	–	–
	Falling number (Hagberg–Perten)	FALLING_N	sec	DIN EN ISO (2009)	Activity of starch decomposition enzymes (amylase) determines the magnitude of the falling number. High values are desired. Low values influence the baking quality by weakening the crumb elasticity of cookies
	Crude grain protein concentration (% of dry matter)	PROTEIN_C	%	ICC 105/2 and DIN EN 15948 (2012)	Protein concentration can be considered to be a quantitative indicator of wheat quality. Decreasing protein content reduces elasticity of gluten and hence the elasticity of the dough
	Sedimentation value (Zeleny index)	SEDIMNT_V	ml	ICC 116/1	An important criterion for the protein quality. Swelling of the gluten fraction of flour in lactic acid solution affects the rate of sedimentation of a flour suspension in the lactic acid medium. Higher gluten content and better gluten quality both give rise to slower sedimentation and higher sedimentation test values
	Hardness	HARDNESS	%	AG Getreide (2016)	Describes the degree of fineness of the flour, assessed by the retention yield of a 75 µm sieve as percent of total flour weight. Flours with medium to high hardness are preferred for bread baking
	Water absorption of the flour (Brabender Farinograph)	WATER_A	%	ICC 115/1	Amount of water held by flour at 14% dry matter as percent of total flour weight. Depends upon the protein concentration and the gluten swelling capacity. Decisive for loaf volume and dough consistency
	Mineral concentration in millstream flour	MINERAL_C	%	ICC 104/1	Quantity of mineral matter in millstream flour as percent to tested flour quantity
	Millstream flour yield	MILLSTR_Y	%	AG Getreide (2016)	Flour yield after six mill streams (one grinding pass and one sifting pass) in a laboratory mill as percent of milled grain
	Mineral value number	MINERAL_V		AG Getreide (2016)	$\frac{{ {\text{\% mineral concentration in millstream flour }}}}{\text{millstream flour yield}} {\text{x }}100, 000$ Closely related to milling yield (type 550). Varieties with low mineral value number are well suited for current milling technology
	Milling yield (type 550)	MILLING_Y	%	AG Getreide (2016)	Millstream flour yield with a standardized mineral concentration of 0.6% as percent of milled grain weight
	Loaf volume (rapid-mix-test)	LOAF_V	ml/100 g	AG Getreideforschung (2007)	Standard baking test with flour type 550. Central quality criterion, measures the volume of bread obtained from 100 g of flour. Used to allocate varieties to quality groups
On-farm	Grain yield at 86% dry matter	GRAIN_Y	dt ha^−1^	Besondere Ernte- und Qualitaetsermittlung (BEE) (2014)	National average yield surveyed from on-farm winter wheat harvests between 1983 and 2014
	Crude grain protein concentration (% of dry matter)	PROTEIN_C	%	As for VCU	As for VCU
	Sedimentation value (Zeleny index)	SEDIMNT_V	ml	As for VCU	As for VCU
	Loaf volume (calculated)	LOAF_V	ml/100 g	Bolling (1969)	Loaf volume is not a laboratory result. It is a calculated variable which predicts loaf volume on the basis of a linear regression function with independent variables protein concentration and sedimentation value. Functions are different for individual quality groups (Bolling 1969; Huesken et al. 2014)

A standard variety stays in VCU trials for about 7.5 years on the average, whereas a candidate varieties’ statutory testing period is 3 years. The data comprised 32 years (1983–2014) and 59–67 different trial sites. The data set was very non-orthogonal, covering only about 1.6% of the possible variety-location-year-combinations.

To avoid biased results, we checked data thoroughly for consistency in structure over time before carrying out analysis. Inconsistent data structures may have occurred due to changes in assessment of a characteristic’s scale of measurement, structure of trial series or laboratory methods. The data were further checked for recording errors and outliers by calculating standardized residuals based on model (1), (2) and (3), as described later in “Statistical analysis”. Observations with standardized residuals greater than ±5.0 were excluded from further analysis. A total number of 56 (0.047%) observations exceeded the threshold and were eliminated.

#### On-farm data

In the German annual national statutory survey of bread cereal quality, about 2000 representative on-farm samples were drawn every year (Huesken et al. 2014). This study uses data collected between 1983 and 2014. The data were made available from the annual survey reports [“Besondere Ernte- und Qualitaetsermittlung (BEE)’’ 2014]. For grain yield, only annual national averages were available from survey reports, covering varieties of all quality grades. Data for grain protein concentration, sedimentation value and expected loaf volume have been reported as variety by year means (Table 1). In this study, we will refer to the variety by year data of these three traits as the on-farm data set. Expected loaf volume is not a laboratory result, it is in fact a calculated variable which predicts loaf volume (Table 1). Additionally, we included the relative sample size as percentage of total sample size for each variety. This measure should relate to the on-farm growing area of the variety. In the annual survey report, only varieties with larger sample sizes were reported. On average, results of 26 varieties were published each year. They cover about 90% of the winter wheat growing area. For this study, however, we eliminated varieties registered in another EU country and varieties which were not quality graded. Expected loaf volume was not calculated for all samples, because the formula is not valid for varieties in quality group C. On theses grounds, we further dropped varieties with C-quality, too. Information was provided on a total of 115 varieties of groups E, A and B. In total, 695 observations were available from these groups. The oldest variety was 1955 for its first year in test and the youngest in 2012. The data set covered about 20% of the possible variety-year-combinations.

#### VCU trial data vs on-farm data

To compare trial and on-farm results, only varieties in quality groups A, B and E were included in a separate VCU trial data set used for comparison, except for grain yield. In the on-farm data set, 56 varieties fall into group A, 43 in group B and 16 in group E, whereas in the VCU trial data set 112 varieties belong to group A, 115 to group B and 40 to group E. 86 varieties were in common. In the survey data, the oldest variety was first assessed in 1955 whereas its first year in the VCU trials was 1963. The average age of varieties in the VCU trial data set was 3.5 years and in on-farm 10.5 years, where the age of a variety is considered as the difference between its actual testing year and its first year in trial. If we consider a variety’s growing area as dominant if it exceeds the 10% threshold of total wheat growing area, then about 9% of candidate varieties tested between 1983 and 2014 became dominating after about 8 years since their first year in trial.

Best local practice in trial management naturally developed over time due to improvement of several factors, like more effective growth regulators and fungicides as well as higher precision of sowing and harvesting technique. It is reasonable to assume that on-farm crop management developed in parallel; however, we are not aware of any studies monitoring long-term changes of on-farm crop management on a national scale.

### Statistical analysis

#### Model for genetic and non-genetic trend

We used the standard three-way model with factors genotype, location and year given by Laidig et al. (2008)

1 $$ y_{ijk} = \mu + G_{i} + L_{j} + Y_{k} + \left( {LY} \right)_{jk} + \left( {GL} \right)_{ij} + \left( {GY} \right)_{ik} + \left( {GLY} \right)_{ijk} , $$

where *y*_*ijk*_ is the mean yield of the *i*th genotype in the *j*th location and *k*th year, *μ* is the overall mean, *G*_*i*_ is the main effect of the *i*th genotype, *L*_*j*_ is the main effect of the *j*th location, *Y*_*k*_ is the main effect of the *k*th year, (*LY*)_*jk*_ is the *jk*th location × year interaction effect, (*GL*)_*ij*_ is the *ij*th genotype × location interaction effect, (*GY*)_*ik*_ is the *ik*th genotype × year interaction effect, and $\left( {GLY} \right)_{ijk}$ is a residual comprising both genotype × location × year interaction and the sampling error arising from sampling the replications. Quality traits assessed on bulked laboratory samples are additionally subject to errors arising from laboratory processing. This model assumes that locations are crossed with years, i.e. at least some locations are used across several years. All effects except *μ*, *G*_*i*_ and *Y*_*k*_ are assumed to be random and independent with constant variance for each effect. Genetic and non-genetic time trend were studied by modelling *G*_*i*_ and *Y*_*k*_ with regression terms for time trends as follows (Laidig et al. 2014; Piepho et al. 2014a):

2 $$ G_{i} = \beta r_{i} + H_{i} , $$

where $\beta$ is a fixed regression coefficient for genetic trend, *r*_*i*_ is the first year in trial for the *i*th variety, and *H*_*i*_ models a random normal deviation of *G*_*i*_ from the genetic trend line, and

3 $$ Y_{k} = \gamma t_{k} + Z_{k} , $$

where $\gamma$ is a fixed regression coefficient for the non-genetic trend, *t*_*k*_ is the continuous covariate for the calendar year and *Z*_*k*_ is a random normal residual. Genetic and non-genetic trends are quantified by the regression coefficients *β* and *γ*, respectively, indicating the yield increase per year measured in the same units as *y*_*ijk*_.

#### Model for overall trend

Overall trend was modelled considering the genotype as nested within years (Laidig et al. 2014). Thus, compared with model (1), for this analysis we dropped effects involving genotypes that are not nested within years, i.e. the effects $G_{i}$ and (*GL*)_*ij*_. The reduced model is given by

4 $$ y_{ijk} = \mu + L_{j} + Y_{k} + \left( {LY} \right)_{jk} + \left( {GY} \right)_{ik} + \left( {GLY} \right)_{ijk} $$

Similarly as in Eq. (3), the year main effect can be modelled as

5 $$ Y_{k} = \phi t_{k} + U_{k} , $$

where* ϕ* is a fixed regression coefficient for overall trend, *t*_*k*_ is the continuous covariate for the calendar year and *U*_*k*_ is a random residual following a normal distribution with zero mean and variance $\sigma_{U}^{2}$. We take the year main effects as fixed to obtain adjusted means for years, representing the overall trend.

#### Performance gain from 1983 to 2014

To quantify the difference in performance levels of individual traits at the beginning and at the end of period studied, we calculated the differences between the overall linear regression estimate in 1983 and 2014 and expressed the difference relative to overall regression estimate at calendar year 1983.

#### Model extension for genetic trend with varieties in quality groups

To study trends in individual groups, we extended Eq. (2) to

6 $$ G_{il} = \beta_{l} r_{i} + H_{il} , $$

where $\beta_{l}$ denotes the fixed regression coefficient for the genetic trend of group *l* = 1,…,*L.*

We further allowed for individual overall means $\mu_{l}$ for groups *l* in model (1).

It is assumed that the non-genetic trend is identical for all groups and that the random effects in models (1), (3) and (6) are homogeneous within and between groups.

#### Model extension for overall trend with varieties in groups

To study overall trends of individual groups, we modified Eq. (5) to

7 $$ Y_{kl} = \phi_{l} t_{k} + U_{kl} , $$

where *Y*_*kl*_ is the main effect of the *k*th year for the *l*th group, $\phi_{l}$ denotes the fixed regression coefficient for the overall trend of the *l*th group, assuming that $U_{lk}$ has homogeneous variances within and between groups.

#### Genetic correlation

We estimated genetic correlation coefficients between traits by a univariate approach (Piepho et al. 2014b):

1. Calculate variance components according to the linear trend model [Eqs. (1–3)] for trait (*p*) and (*q*) and for the difference between both traits.
2. Compute covariances between the genotypic effects $H_{i}$ [Eq. (2)] from variance components obtained from univariate models using the equation
  8 $$ \text{var} (H^{(p)}_{i} - H^{(q)}_{i} ) = \text{var} (H^{(p)}_{i} ) + \text{var} (H^{(q)}_{i} ) - 2\text{cov} (H^{(p)}_{i} ,H^{(q)}_{i} ) \Leftrightarrow \text{cov} (H^{(p)}_{i} ,H^{(q)}_{i} ) = \frac{{\text{var} (H^{(p)}_{i} ) + \text{var} (H^{(q)}_{i} ) - \text{var} (H^{(p)}_{i} - H^{(q)}_{i} )}}{2} $$
3. Use variances from Eq. (2) and covariance from Eq. (8) to calculate the genetic correlation coefficient *ρ*_g_.

#### Phenotypic correlation

To evaluate phenotypic correlation between quality traits, we considered effects for genotype and year to be fixed in model (1) and then calculated least square means for genotypes. We expressed correlation between traits by the Pearson correlation coefficient of least square means for genotype.

#### Weighted analysis of on-farm data

For data from national survey, variety by year means and the relative sample size were available. We, therefore, adjusted models (1) and (4) developed above for VCU trial data analogously by dropping effects for location, location by year and by variety. For the reduced models, we applied a weighted mixed model analysis using the relative sample size as weight to take into account different growing areas of varieties. Varieties with higher growing areas get more influence on the estimates than varieties with lower areas. In the analysis of VCU trial data, however, each variety was equally weighted.

#### Graphical displays

A fixed categorical effect $C_{p}$ for time class $p = 1, \ldots ,P$ will be introduced, where $P$ is the number of levels of the time variable $r_{i}$ (a variety’s first year in trial). Each time class is represented by at least one genotype. Then, the genetic effect can be modelled as

9 $$ G_{i} = C_{p} + H'_{i} , $$

where $H'_{i}$ is the random deviation from categorical effect $C_{p}$. We compute adjusted means (least square means) for $C_{p}$ and plot them against first year of testing ($r_{i}$). Eq. (9) is applied analogously if quality groups are considered as described in model (6).

The plots used based on the proposed models are described in Table 2.

**Table 2:** Graphical displays of VCU and of on-farm results

Description	Ordinate	Abscissa	Equations used	Figures
Visible genetic trend	Adj. genotype class means *C*_*p*_	Year of first testing *r*_*i*_	Equation (9) inserted in baseline model (1) keeping *C*_*p*_ and *Y*_*k*_ fixed	Figure 2: all quality groups; Figure 3: quality groups E, A, B
Visible agronomic trend	Adj. year means for *Y*_*k*_	Calendar (harvest) year *t*_*k*_	Model (1) keeping *G*_i_ and *Y*_*k*_ fixed	Figure 3: quality groups E, A, B
Visible overall trend	Adj. year means for *Y*_*k*_	Calendar (harvest) year *t*_*k*_	Model (4)	Figure 2: all quality groups; Figure 3: quality groups E, A, B
Genotype by year plots	Adj. genotype means *G*_*i*_	Year of first testing *r*_*i*_	Model (1) keeping effects for genotypes *G*_*i*_ and years *Y*_*k*_ fixed	Figure 4
Correlation plots	Adj. genotype means *G*_*i*_	Adj. genotype means *G*_*i*_	Model (1) keeping effects for genotypes *G*_*i*_ and years *Y*_*k*_ fixed	Figures 6, 7, 8, S2

On-farm data are based on variety by year means; equations are applied analogously (see Materials and methods)

## Results

### Performance progress in VCU trials and on-farm

#### VCU trials including all quality groups

**Fig. 2:**
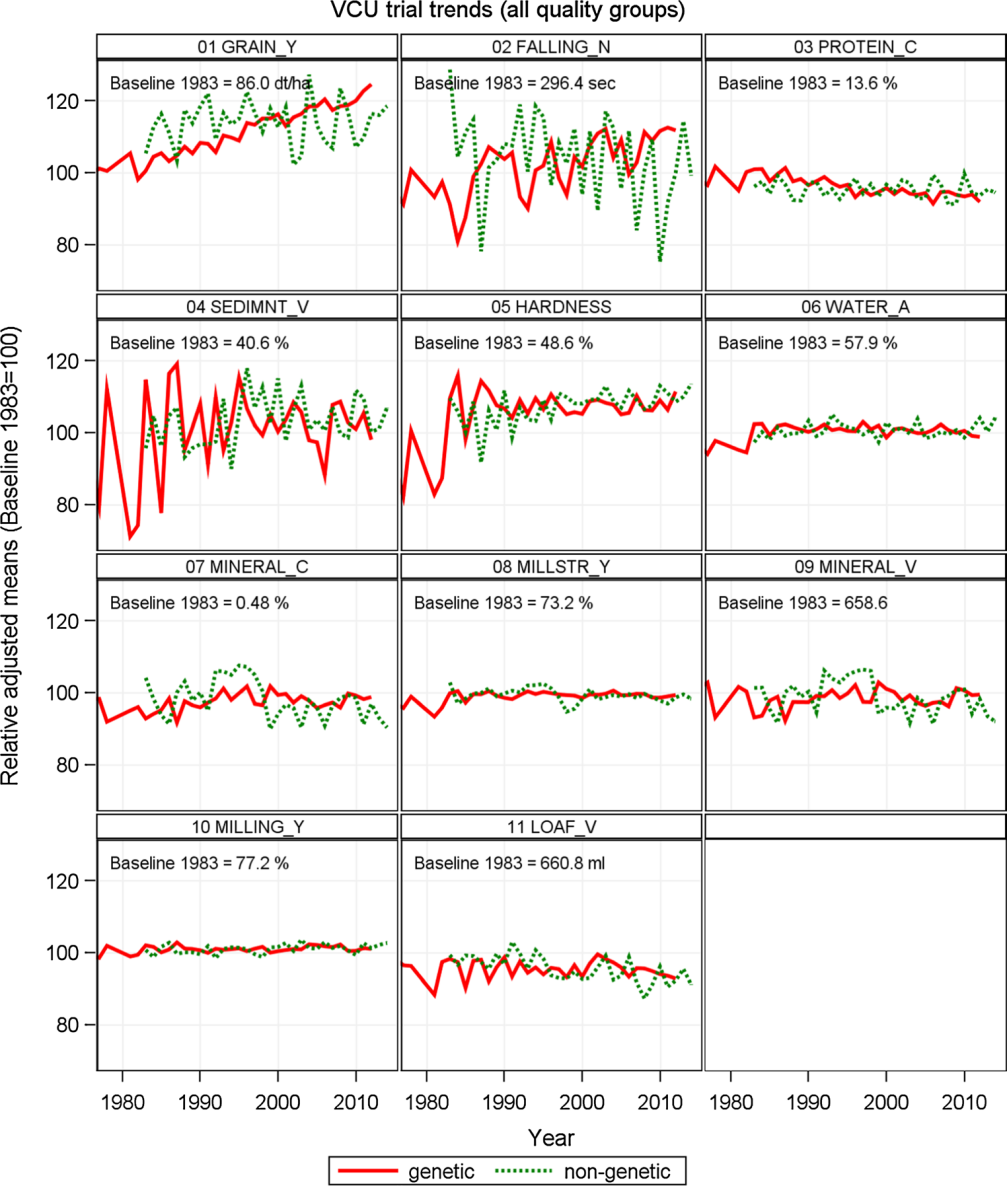
Relative adjusted means as percent of 1983 baseline. Genetic: variety group means [effect *C*_*p*_ in Eq. (9)]. Non-genetic: year means [Eq. (1), using Eq. (9) to model *G*_*i*_]. *GRAIN*_*Y* grain yield, *FALLING*_*N* falling number, *PROTEIN*_*C* crude protein concentration, *SEDIMNT*_*V* sedimentation value, *HARDNESS* hardness, *WATER_A* water absorption, *MINERAL*_*C* mineral concentration, *MILLSTR*_*Y* millstream flour yield, *MINER*AL_*V* mineral value number, *MILLING*_*Y* milling yield, *LOAF*_*V* loaf volume

In Table 3, we compare trends representing progress achieved in VCU trials and on-farm between 1983 and 2014. Genetic, non-genetic and overall trends are displayed in Fig. 2 for VCU trials.

**Table 3:** Performance levels 1983 and 2014, difference in performance levels between 2014 and 1983, and estimates of linear regression coefficients for trends

Traits		Source	Overall regression estimates				Estimates of linear trends								
							Genetic			Non-genetic			Overall		
Short name	Unit		1983	2014	Diff.	%Diff.	Absolute	SE	%	Absolute	SE	%	Absolute	SE	%
GRAIN_Y	dt ha^−1^	Trial	86.0	106.4	20.4^***^	23.8	0.559^***^	0.040	0.65	0.075	0.112	0.09	0.659^***^	0.110	0.77
		Trial^b^	85.5	105.6	20.1^***^	23.5	0.541^***^	0.041	0.63	0.074	0.111	0.09	0.647^***^	0.108	0.76
		On-farm^a^	60.9	80.1	19.2^***^	31.6							0.620^***^	0.086	1.02
FALLING_N	s	Trial	296.4	313.7	17.3	5.8	1.375^**^	0.372	0.46	−0.930	0.736	−0.31	0.557	0.702	0.19
PROTEIN_C	%	Trial	13.6	12.5	−1.1^***^	−8.0	−0.028^***^	0.004	−0.21	−0.006	0.008	−0.05	−0.035^***^	0.007	−0.26
		Trial^b^	13.7	12.7	−1.0^***^	−7.4	0.025^***^	0.005	−0.19	−0.007	0.008	−0.05	−0.033^***^	0.007	−0.24
		On-farm^b^	12.9	13.1	0.2*	1.5	0.016*	0.007	0.13	−0.010	0.008	−0.07	0.008^*^	0.003	0.05
SEDIMNT_V	ml	Trial	40.6	43.8	3.2	7.9	−0.006	0.087	−0.02	0.072	0.067	0.18	0.103	0.066	0.26
		Trial^b^	43.0	47.0	4.0	9.3	0.006	0.085	0.01	0.081	0.073	0.19	0.129	0.073	0.30
		On-farm^b^	35.6	51.7	16.1^***^	45.4	0.307^***^	0.083	0.86	0.117	0.063	0.33	0.542^***^	0.045	1.46
HARDNESS	%	Trial	48.6	55.1	6.5^*^	13.4	0.064^*^	0.032	0.13	0.128^**^	0.041	0.26	0.002^*^	0.210	0.43
WATER_A	%	Trial	57.9	58.6	0.7	1.2	−0.011	0.017	−0.02	0.026	0.023	0.05	0.022	0.022	0.04
MINERAL_C	%	Trial	0.48	0.46	−0.03	−5.2	0.0004	0.0002	0.07	−0.0011^*^	0.0005	−0.24	−0.0008	0.0005	−0.17
MILLSTR_Y	%	Trial	73.2	72.2	−1.0	−1.4	0.010	0.013	0.01	−0.048	0.026	−0.07	−0.033	0.027	−0.05
MINERAL_V		Trial	658.6	632.8	−25.9	−3.9	0.358	0.340	0.05	−1.111	0.626	−0.17	0.607	0.169	−0.13
MILLING_Y	%	Trial	77.2	79.1	1.8^**^	2.4	0.010	0.014	0.01	0.046^*^	0.022	0.06	0.019^**^	0.002	0.08
LOAF_V	ml	Trial	660.8	604.3	−56.5^***^	−8.5	−0.344	0.361	−0.05	−1.602^***^	0.419	−0.24	−1.822^***^	0.432	−0.28
		Trial^b^	667.7	621.1	−46.6^***^	−7.0	−0.088	0.315	−0.01	−1.574^***^	0.421	−0.24	−1.502^***^	0.412	−0.23
		On-farm^b^	642.4	695.8	53.4^***^	8.3	1.209^**^	0.404	0.19	0.025	0.346	0.00	1.810^***^	0.215	−0.27

Percent trends (%) are relative to 1983 performance levels

*SE* standard errors of regression coefficients, *GRAIN*_*Y* grain yield, *FALLING*_*N* falling number, *PROTEIN*_*C* crude protein concentration, *SEDIMNT*_*V* sedimentation value, *HARDNESS* hardness, *WATER*_*A* water absorption, *MINERAL*_*C* mineral concentration, *MILLSTR*_*Y* millstream flour yield, *MINERAL*_*V* mineral value number, *MILLING*_*Y* milling yield, *LOAF*_*V* loaf volume, on-farm loaf volume (calculated)

* Significant at 5% level

** Significant at 1% level

*** Significant at 0.1% level

^a^On-farm, all quality groups (national average yield)

^b^Trials and on-farm, only quality groups E, A and B

As shown in Table 3, a significant gain of 23.8% (20.4 dt ha^−1^) was achieved in grain yield, in hardness 13.4% (6.5% absolute change) and in milling yield 2.4% (1.8% absolute change) relative to 1983. But significant losses in protein concentration of −8.0% (−1.1% absolute change) and in loaf volume of −8.5% (−56.5 ml) relative to 1983 were found. Only moderate gains in falling number and sedimentation value and moderate losses in mineral concentration and mineral value were found (Table 3), though not significant. Gain in grain yield as well as the loss in protein concentration is almost completely genetically driven at a rate of 0.65% (0.559 dt ha^−1^ year^−1^), and −0.21% (−0.028% year^−1^ absolute trend) p. a. since 1983, respectively. The significant positive genetic trend in falling number of 0.46% (1.375 s) p. a. was nearly compensated by a negative, however, not significant non-genetic component of −0.31% (−0.930 s) p. a. Non-genetic trends dominate in hardness with 0.26% (0.128% year^−1^ absolute trend) and in loaf volume with −0.24% (−1.602 ml year^−1^) p.a. relative to 1983 (Table 3; Fig. 2). In general, we found a large gain in grain yield, but a considerable reduction in protein concentration. And in both traits this trend is mainly genetically driven. For other quality traits, partially positive and negative trends occurred.

#### VCU trials excluding quality group C

To make VCU trial results comparable with on-farm results for grain yield, protein concentration, sedimentation value and loaf volume, we dropped all 49 C-group varieties from trial data set and presented results in the second row of the respective traits in Table 3.

Results, as compared with the complete data set, indicated that grain yield level was only slightly reduced and protein level only slightly elevated, as shown in Table 3. Levels of sedimentation value and loaf volume were more clearly raised.

#### VCU trial vs on-farm

**Fig. 3:**
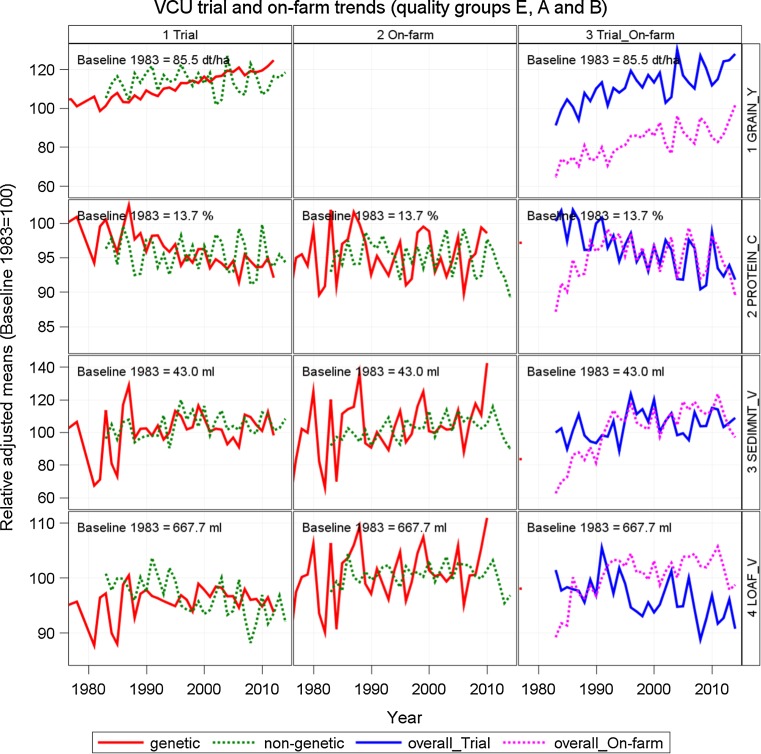
Relative adjusted means as percent of 1983 baseline. Genetic and non-genetic trends from trial data are displayed in *column 1* and from on-farm data in *column 2*, and overall trends from trial and on-farm data in *column 3*. Included are quality groups A, B and E. On-farm trend for grain yield (*column 3*) comprises all varieties grown. Genetic: variety group means [effect *C*_*p*_ in Eq. (9)]. Non-genetic: year means [Eq. (1), using Eq. (9) to model *G*_*i*_]. Overall Trial, overall On-farm: overall year means [*Y*_*k*_ in Eq. (4)]. *GRAIN*_*Y* grain yield, *FALLING*_*N* falling number, *PROTEIN*_*C* crude protein concentration, *SEDIMNT*_*V* sedimentation value, *HARDNESS* hardness, *WATER*_*A* water absorption, *MINERAL*_*C* mineral concentration, *MILLSTR*_*Y* millstream flour yield, *MINERAL*_*V* mineral value number, *MILLING*_*Y* milling yield, *LOAF*_*V* loaf volume, on-farm loaf volume (calculated)

Besides the results for VCU trials with the complete data set and the VCU data set reduced by C-group varieties in Table 3, we added a third row with on-farm results to compare the progress achieved for grain yield, protein concentration, sedimentation value and loaf volume. Trends of both data sets may be seen in Fig. 3. On-farm grain yield data were available only as national year means including all varieties. We compared gain with VCU results including C-group varieties only for this trait.

We observed parallel progress in VCU trial and on-farm yield (Fig. 3). Relative gain in on-farm grain yield was considerably higher (31.6%, 19.2 dt ha^−1^) than for trials (23.8%, 20.4 dt ha^−1^) due to mean yields on-farm being lower by about 25 dt ha^−1^ (Table 3).

We found considerable differences between trial and on-farm data in the three most important traits for wheat baking quality (Table 3; Fig. 3). On-farm protein concentration slightly increased by 1.5% (0.2% absolute change) relative to 1983, whereas the loss in trials of −7.4% (−1.0% absolute change) was remarkably pronounced. The corresponding genetic trends for this trait were significant for both data sets, but with inverse signs. The gain observed for on-farm sedimentation value [45.4% (16.1 ml)] exceeded the gain in trials [9.3% (4.0 ml)] by the factor 4 relative to 1983. A rather contrasting picture emerged for the trends of loaf volume in both data sets. The on-farm gain was 8.3% (53.4 ml) generated by a significant genetic trend of 0.19% (1.209 ml year^−1^) p. a., whereas the loss observed in the VCU trials of −7.0% (−46.6 ml year^−1^) was highly significant, but was generated by a strong and highly significant non-genetic trend of −0.24% (−1.574 ml year^−1^) p. a. (Table 3; Fig. 3).

#### Trials vs on-farm by quality groups

**Fig. 4:**
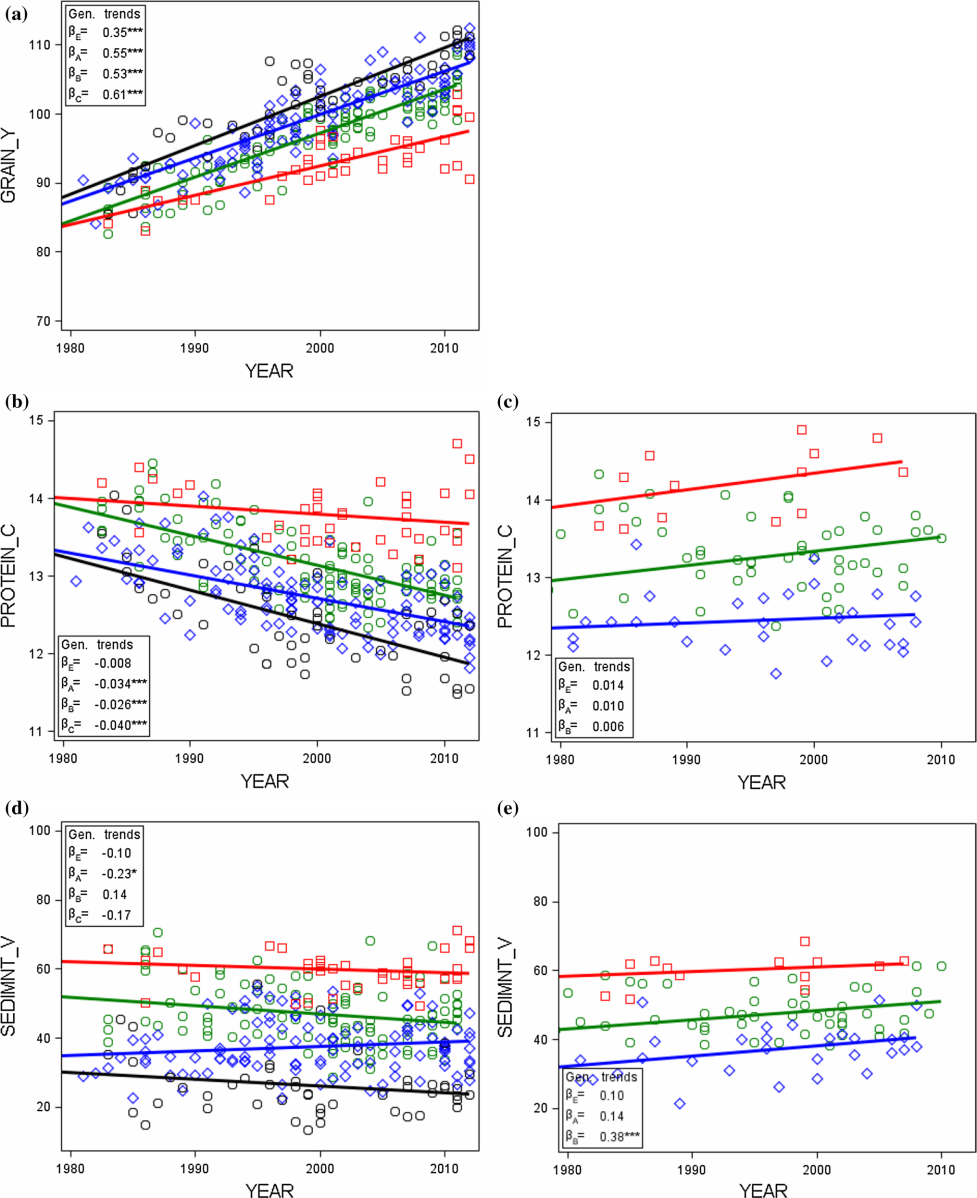

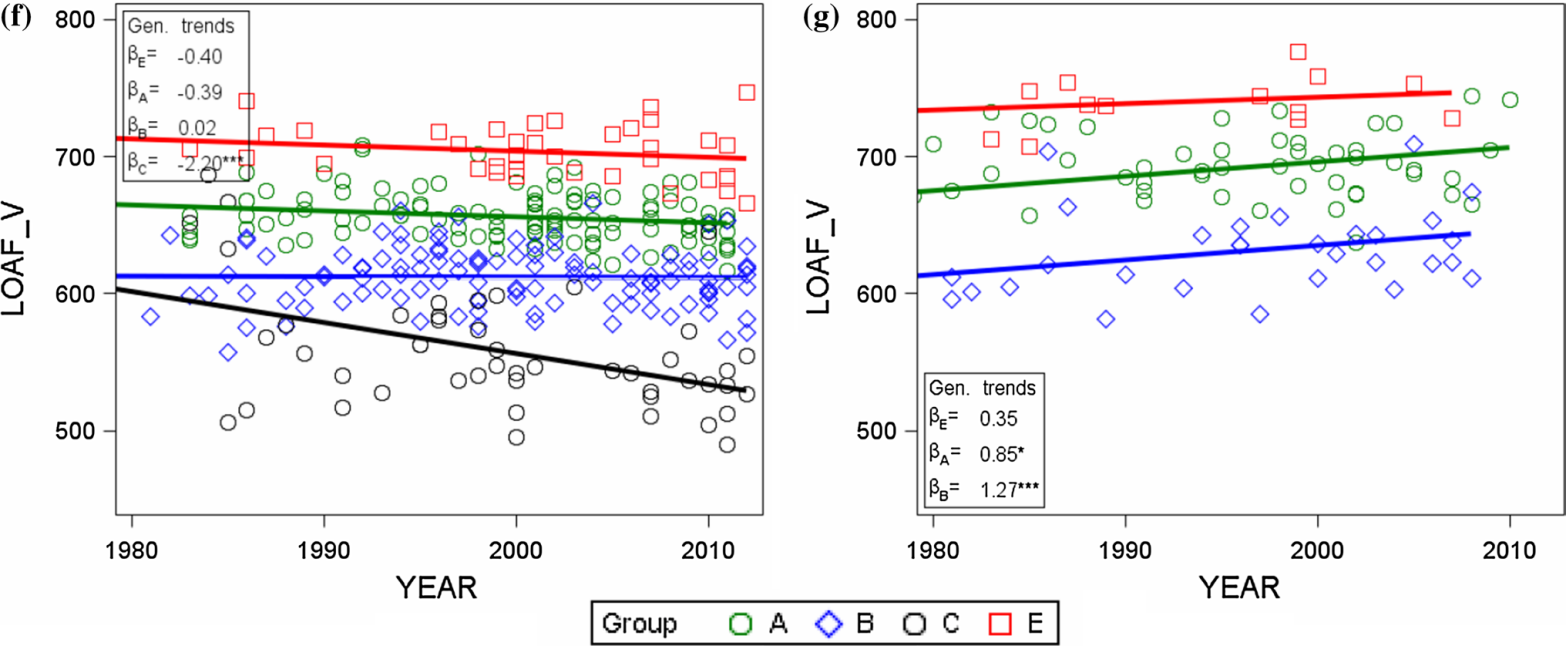
VCU trial (*left column*) and on-farm (*right column*) adjusted means [*G*_*i*_ in Eq. (1)] by quality groups (grades in descending order are *E* elite wheat, *A* quality wheat, *B* bread wheat, *C* others) plotted against first year in trial with group regression lines for(**a**) grain yield, (**b**) and (**c**) crude protein concentration, (**d**) and (**e**) sedimentation value, and (**e**) and (**f**) loaf volume. Regression lines for quality groups are plotted to display genetic trends as indicated in *inset boxes* (Table 4). *β*_*E*_, *β*_*A*_, *β*_*B*_, *β*_*C*_ genetic trends for quality groups [Eq. (1) using Eq. (6)]. *GRAIN*_*Y* grain yield, *PROTEIN*_*C* crude protein concentration, *SEDIMNT*_*V* sedimentation value, *LOAF*_*V* loaf volume, on-farm loaf volume (calculated)

Individual quality group means, regression coefficients for genetic and non-genetic trends and F values for a test of heterogeneous regression lines of genetic trends are listed in Table 4 for VCU trial and on-farm data. A group-wise representation of adjusted variety means plotted against their first year in trial is shown in Fig. 4.

**Table 4:** Comparison of VCU trial and on-farm data by quality groups

Traits	Unit	Qualitiy group	Overall regression estimates				Estimates of linear trends				Test for heterogeneous linear genetic trends	
			1983		2014		Genetic		Non-genetic		*F* value	
			VCU	On-farm	VCU	On-farm	VCU	On-farm	VCU^a^	On-farm^b^	VCU^a^	On-farm^b^
GRAIN_Y	dt ha^−1^	*E*	81.9		97.7		0.353^***^		0.133		6.43^***^	
		*A*	84.4		106.0		0.547^***^					
		*B*	86.4		109.2		0.533^***^					
		*C*	89.1		111.5		0.614^***^					
PROTEIN_C	%	*E*	14.3	13.6	13.6	14.7	−0.008	0.014	−0.009	−0.005	5.92^***^	0.75
		*A*	14.0	13.2	12.6	13.1	−0.034^***^	0.010				
		*B*	13.4	12.6	12.2	12.2	−0.026^***^	0.006				
		*C*	13.3		11.8		−0.040^***^					
SEDIMNT_V	ml	*E*	63.2	53.6	60.1	66.3	−0.101	0.165	0.062	0.140	3.62^*^	3.58^*^
		*A*	49.6	42.9	45.5	51.3	−0.225^*^	0.141				
		*B*	33.3	28.8	40.5	41.5	0.141	0.376^***^				
		*C*	27.8		24.5		−0.185					
VOLUME_Y	ml	*E*	737.6	716.4	678.1	764.9	−0.404	0.353	−1.664^***^	−0.046	8.85^***^	1.11
		*A*	688.9	677.8	622.2	703.6	−0.388	0.851^*^				
		*B*	638.6	615.9	586.7	632.7	0.018	1.269^***^				
		*C*	620.0		505.9		−2.203^***^					

Performance levels 1983 and 2014 are based on overall regression estimate

Number of varieties in quality groups: *E* = 40, *A* = 112, *B* = 115, *C* = 49 in trials; *E* = 16, *A* = 56, *B* = 43 on-farm. 86 varieties were identical in both data sets

*GRAIN*_*Y* grain yield, *PROTEIN*_*C* crude protein concentration, *SEDIMNT*_*V* sedimentation value, *LOAF*_*V* loaf volume, on-farm loaf volume (calculated)

* Significant at 5% level

** Significant at 1% level

*** Significant at 0.1% level

^a^This value refers to quality groups *E*, *A*, *B* and *C*

^b^This value refers to quality groups *E*, *A* and *B*

*F* tests for heterogeneous linear genetic trends indicate that for all VCU trial traits there are significantly different group-wise slopes, whereas for on-farm results significant differences exist only for protein concentration and sedimentation value (Table 4). This discrepancy may be explained partially by the fact that the significance test for the trial data is based on more observations. Genetic progress in trial grain yield of group E is lower (*β*_*E*_ = 0.353 dt ha^−1^ year^−1^) than of groups A, B, C with rates above 0.5 dt ha^−1^ year^−1^ (Table 4; Fig. 4a). A similar, but reversed pattern was found for protein concentration. For sedimentation value and loaf volume, our results indicate negative, but non-significant slopes, except for sedimentation value of group A.

On-farm results indicate significantly heterogeneous genetic trends for sedimentation value. Genetic trend of protein concentration shows non-significant positive rates (Table 4; Fig. 4c).

A principal difference between VCU trials and on-farm results became visible: genetic trends for protein concentration, sedimentation value and loaf volume for VCU trial data are decreasing, whereas for on-farm data they are increasing (Fig. 4).

### Genotype, environment and genotype by environment interaction in VCU trial data

Estimates of long-term variance components may be biased if time trends are present in random effects. As previously shown in our model (1), genotypic and year effects contain linear trends. Therefore, we have taken into account a linear trend in the genetic effects by $G_{i} = \beta r_{i} + H_{i}$ (Model 2) and for the year effect by $Y_{k} = \gamma t_{k} + Z_{k}$ (Model 3). Variance components for the genotypic effect *H*_*i*_ and the year effect *Z*_*k*_ are then random deviations from linear trends.

**Fig. 5:**
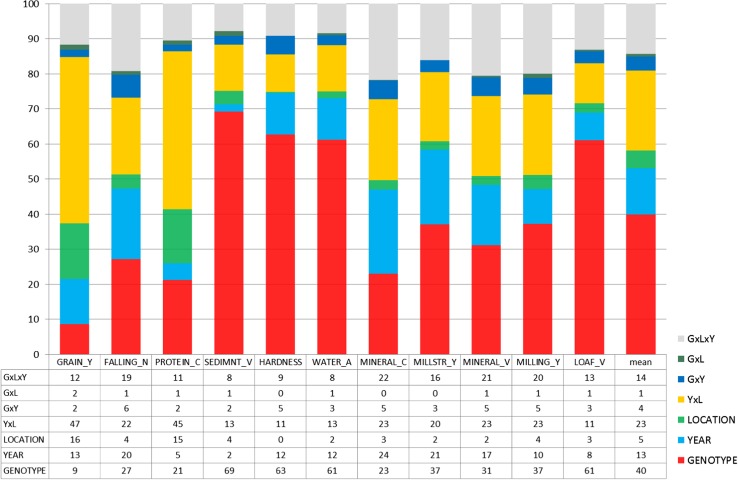
Sources of variation of grain and quality traits from VCU trials (all quality groups) after elimination of genetic and non-genetic trends as percentage of total variability [Eq. (1), using (2) and (3)]. The rightmost column “mean” represents the average over traits in order to give an orientation as to the relative magnitude of individual components. *GRAIN*_*Y* grain yield, *FALLING*_*N* falling number, *PROTEIN*_*C* crude protein concentration, *SEDIMNT*_*V* sedimentation value, *HARDNESS* hardness, *WATER*_*A* water absorption, *MINERAL*_*C* mineral concentration, *MILLSTR*_*Y* millstream flour yield, *MINERAL*_*V* mineral value number, *MILLING*_*Y* milling yield, *LOAF*_*V* loaf volume

It is useful and illustrative to express variance components as percentage of their total sum (Fig. 5). Due to the large data set, all non-zero variance component estimates turned out to be significantly different from zero with *p* < 0.01. The most important component is the genotypic variance. On the average, 40% of the total variance is accounted for by genotypic variation. The range for genotypic variation of sedimentation value, hardness, water absorption and loaf volume was high (60–70%), medium for millstream yield, mineral value and milling yield (30–40%) and low for falling number, protein concentration and mineral value (21–30%). A remarkably low genotypic influence of 9% was found for grain yield.

The mean for environmental variation (year, location and year by location) of 41% was only slightly larger than the genotypic variation (40%). The year by location interaction was the dominating environmental effect (23%). The influence of year (13%) was more than twice as large as that of location (5%). On closer examination, considerable differences exist between individual traits. Environmental effects caused 76% of the total variation for grain yield, followed by protein concentration (65%) and mineral concentration (50%). Low variability across environments was observed for sedimentation value (21%), loaf volume (22%) and hardness (23%). When considering the relation of the year and location component, Fig. 5 shows that for protein concentration year to year variation is remarkably low (5%) as compared to variation caused by location (15%). However, for grain yield the influence of locations (16%) is only slightly larger than for years (13%). For all other traits, variation due to years is greater than for locations, except for sedimentation value (year 2% and location 4%).

The results clearly show that for the traits influencing baking quality, except protein concentration, genotypic variation accounts for more than 60% of total variability and that years are more important than locations to explain variation.

### Phenotypic and genotypic correlation in VCU trial and on-farm data

#### VCU trials

Results in Table 5 indicate that phenotypic correlation coefficients *ρ*_p_ tend to be smaller than corresponding genotypic correlation coefficients *ρ*_g_, especially for grain yield, protein concentration, and falling number.

**Table 5:** Correlations between traits from VCU trials

	GRAIN_Y	FALLING_N	PROTEIN_C		SEDIMNT_V		HARDNESS		WATER_A		MINERAL_C		MILLST_Y		MINERAL_V		MILLNG_Y		VOLUM_Y	
Mean	97.9	308.9	13.0		41.9		52.1		58.2		0.5		72.7		650.3		78.0		630.8	
SD	6.77	43.12	0.62		12.67		4.27		2.44		0.02		1.68		39.11		1.74		49.73	
Min.	78.8	146.6	11.5		13.3		37.3		51.5		0.4		66.8		542.2		71.9		490.1	
Max.	112.5	399.1	14.7		71.1		64.5		65.6		0.5		76.3		749.0		82.9		746.8	
Traits		Correlation coefficients *ρ*																		
GRAIN_Y	1																			
	1																			
FALLING_N	0.08^ns^	1																		
	−0.36	1																		
PROTEIN_C	−0.77	0.16	1																	
	−0.84	0.39	1																	
SEDIMNT_V	−0.42	0.39	0.67		1															
	−0.73	0.45	0.76		1															
HARDNESS	−0.15	0.34	0.35		0.61		1													
	−0.44	0.34	0.47		0.63		1													
WATER_A	−0.31	0.17	0.45		0.53		0.64		1											
	−0.49	0.22	0.49		0.53		0.67		1											
MINERAL_C	0.18	−0.06^ns^	−0.28		−0.21		0.01^ns^		0.07		1									
	0.20	−0.20	−0.27		−0.23		−0.01^ns^		0.08^ns^		1									
MILLSTR_Y	−0.06^ns^	0.22	0.15		0.38		0.53		0.16^ns^		−0.15		1							
	−0.20	0.24	0.23		0.40		0.55		0.17^ns^		−0.20		1							
MINERAL_V	0.18	−0.14^ns^	−0.30		−0.33		−0.20		−0.01		0.92		−0.52		1					
	0.23	−0.19	−0.33		−0.35		−0.23		−0.02^ns^		0.92		−0.57		1					
MILLING_Y	0.01^ns^	0.16	0.10^ns^		0.24		0.15		−0.13^ns^		−0.72		0.69		−0.89		1			
	−0.10^ns^	0.16	0.16		0.25		0.16		−0.13^ns^		−0.74		0.71		−0.91		1			
VOLUME_Y	−0.46	0.36	0.67		0.77		0.52		0.50		−0.18		0.31		−0.28		0.16		1	
	−0.72	0.44	0.75		0.79		0.55		0.52		−0.26		0.33		−0.29		0.17		1	

*Mean* average over adjusted variety means (*n* = 316). *SD* standard deviation, *Min*.: smallest variety mean, *Max*.: Largest variety mean. *Upper value*: Phenotypic correlation coefficient *ρ*_p_. *Lower value*: Genetic correlation coefficient *ρ*_g_. Categorization: 0.25 < |*ρ*| < 0.45 weak, 0.45 ≤ |*ρ*| < 0.65 moderate, 0.65 ≤|*ρ*| < 0.85 strong, 0.85 ≤|*ρ*| very strong

*ns* not significant different from zero at 1% level

*GRAIN*_*Y* grain yield, *FALLING*_*N* falling number, *PROTEIN*_*C* crude protein concentration, *SEDIMNT*_*V* sedimentation value, *HARDNESS* hardness, *WATER*_*A* water absorption, *MINERAL*_*C* mineral concentration, *MILLSTR*_*Y* millstream flour yield, *MINERAL*_*V* mineral value number, *MILLING*_*Y* milling yield, *LOAF*_*V* loaf volume

In general, grain yield is negatively correlated with protein concentration and protein-related quality traits, whereas protein concentration is positively correlated with other quality traits.

Grain yield is most highly negatively associated with protein concentration (*ρ*_p_ = −0.77, *ρ*_g_ = −0.84).

A likewise strong negative genetic relation was found for grain yield with sedimentation value (*ρ*_g_ = −0.73) and with loaf volume (*ρ*_g_ = −0.72), but not phenotypically. Falling number was only weakly associated with all other traits. As expected, protein concentration correlates strongly and positively with sedimentation value (*ρ*_p_ = 0.67, *ρ*_g_ = 0. 76) and with loaf volume (*ρ*_p_ = 0.67, *ρ*_g_ = 0.75). Sedimentation value is strongly associated with loaf volume (*ρ*_p_ = 0.77, *ρ*_g_ = 0.79), but only moderately with hardness and water absorption (Table 5). Hardness correlates moderately with water absorption, millstream yield, and loaf volume (Table 5). The flour traits: mineral concentration, millstream yield, mineral value and milling yield are closely inter-related by nature, but not so for millstream yield and mineral concentration. Mineral value correlates strongly positively with mineral concentration and negatively with millstream yield due to their functional relationship.

**Fig. 6:**
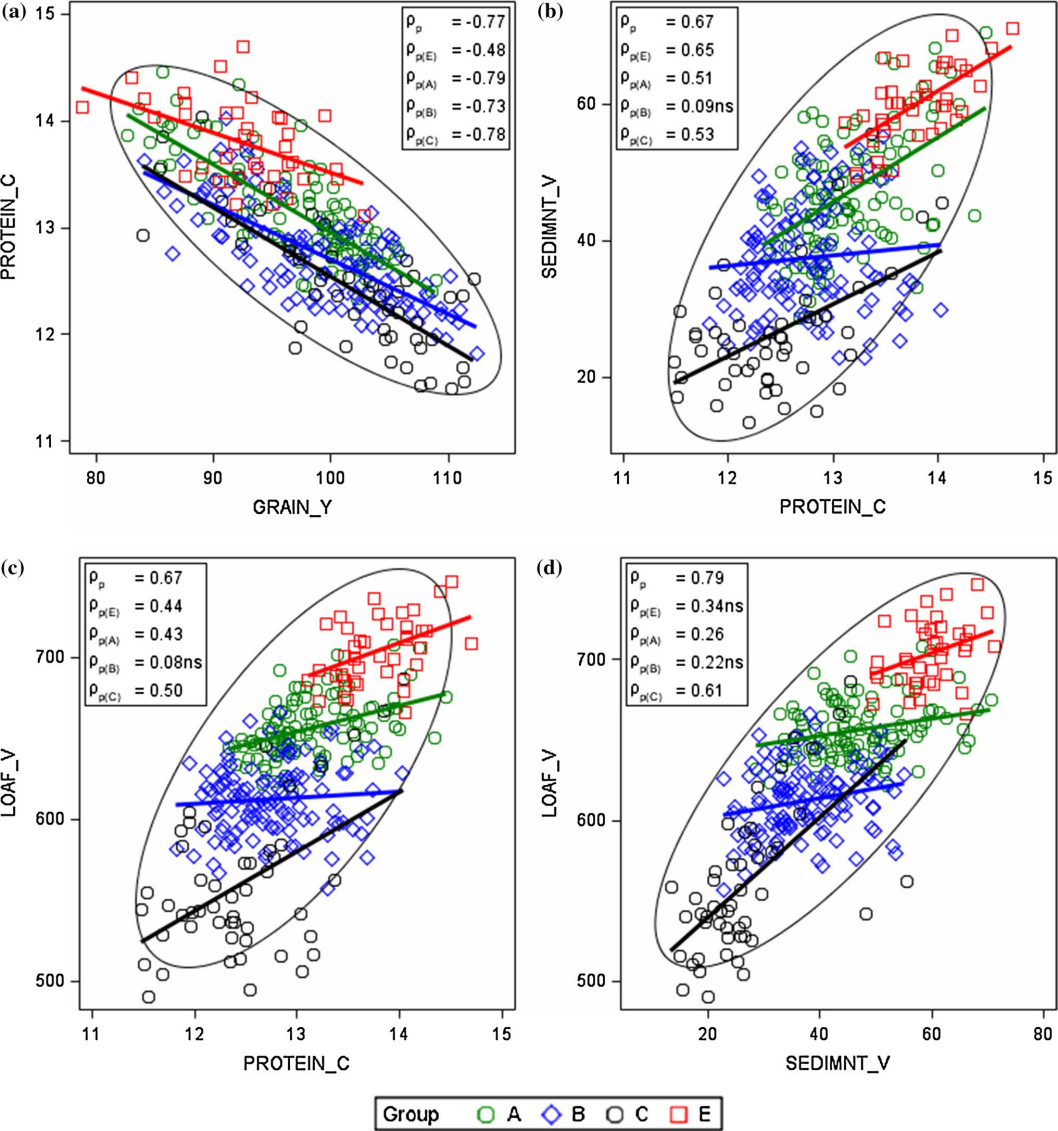
Phenotypic correlation of adjusted variety means [*G*_*i*_ in Eq. (1)] for quality traits from VCU trials. Quality groups with grades in descending order are *E* elite wheat, *A* quality wheat, *B* bread wheat, *C* others. *ρ*_*p*_ phenotypic correlation coefficient over all varieties; *ρ*_*p*(.)_ phenotypic correlation coefficients within groups. *ns* not significant different from zero at 1% level. *GRAIN*_*Y* grain yield, *PROTEIN*_*C* crude protein concentration, *SEDIMNT*_*V* sedimentation value, *LOAF*_*V* loaf volume

In Fig. 6 and in Electronic Appendix Fig.S1, we plotted correlation diagrams of adjusted variety means for selected traits and additionally we marked the varieties according to their quality groups. Phenotypic correlation coefficients over all varieties *ρ*_p_ and coefficients within groups *ρ*_p(.)_ are shown inside boxes in Fig. 6. Group-wise regression lines were drawn to depict the dependence between pairs of traits within groups, and highlight differences between groups. Correlation diagrams in Fig. 6 show that generally (1) coefficients within groups are mostly of the same sign compared to overall correlation, however, of lower magnitude, (2) varieties of groups E and A are less dispersed than those of group B and C, and (3) varieties of group B have smaller correlation coefficients than other groups. It should be noted that the very strong inverse relation between grain yield and protein concentration (*ρ*_p_ = −0.77) also holds for groups A (*ρ*_p(A)_ = −0.79), B (*ρ*_p(B)_ = −0.73) and C (*ρ*_p(C)_ = −0.78), and to a lesser extend for E (*ρ*_p(E)_ = −0.48), as shown by Fig. 6a. For hardness and milling yield, there is apparently no association within nor over groups (Electronic Appendix Fig. S2c), whereas the strong overall relation between hardness and loaf volume was not found for the correlation within groups (Electronic Appendix Fig. S2g).

#### On-farm

**Fig. 7:**
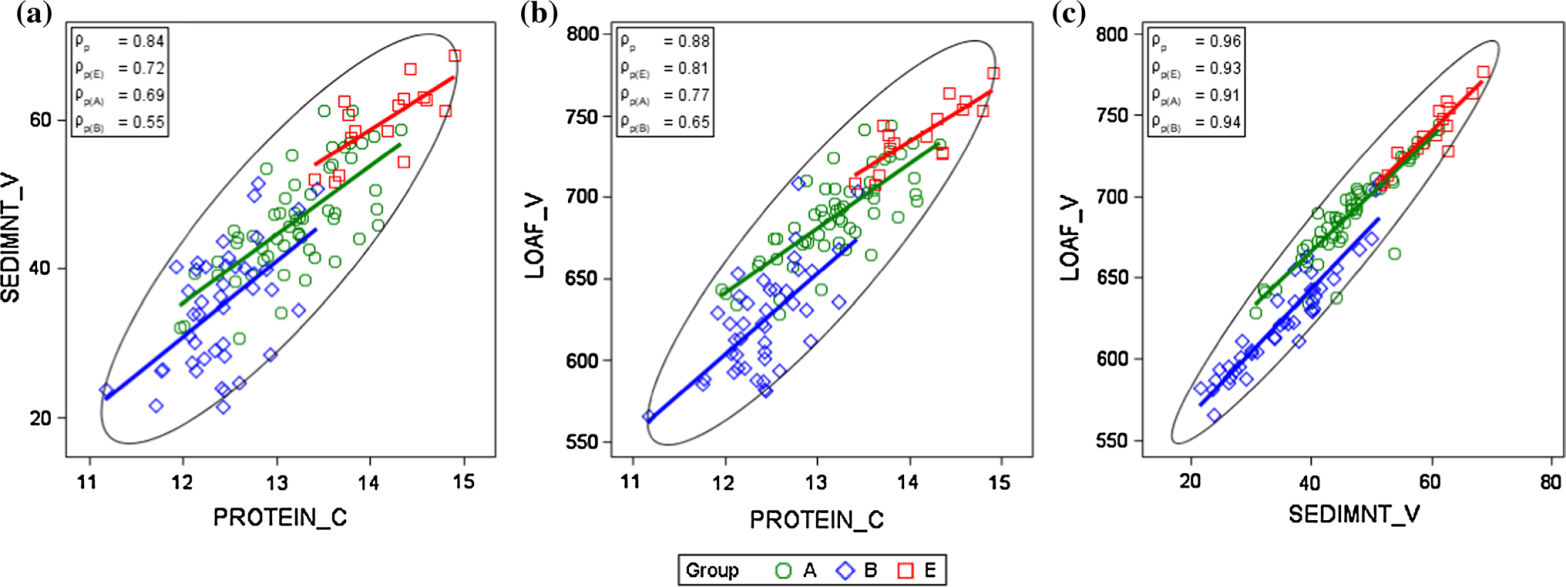
Correlation of adjusted variety means [*G*_*i*_ in Eq. (1)] for quality traits from on-farm data (annual national survey). Quality groups with grades in descending order are *E* elite wheat, *A* quality wheat, *B* bread wheat, *C*. *ρ*_*p*_ phenotypic correlation coefficient over all varieties, *ρ*_*p*(.)_ phenotypic correlation coefficients within groups. *PROTEIN*_*C* crude protein concentration, *SEDIMNT*_*V* sedimentation value, *LOAF*_*V* on-farm loaf volume (calculated)

In Fig. 7a–c, we illustrate the phenotypic relation of quality traits from our on-farm results, which are markedly stronger than the corresponding VCU trial coefficients (Fig. 6b–d). Associations of protein concentration with sedimentation value (*ρ*_p_ = 0.84), protein concentration with loaf volume (*ρ*_p_ = 0.88), and of sedimentation value with loaf volume (*ρ*_p_ = 0.96) were very strong (Fig. 7).

#### VCU trials vs on-farm

**Fig. 8:**
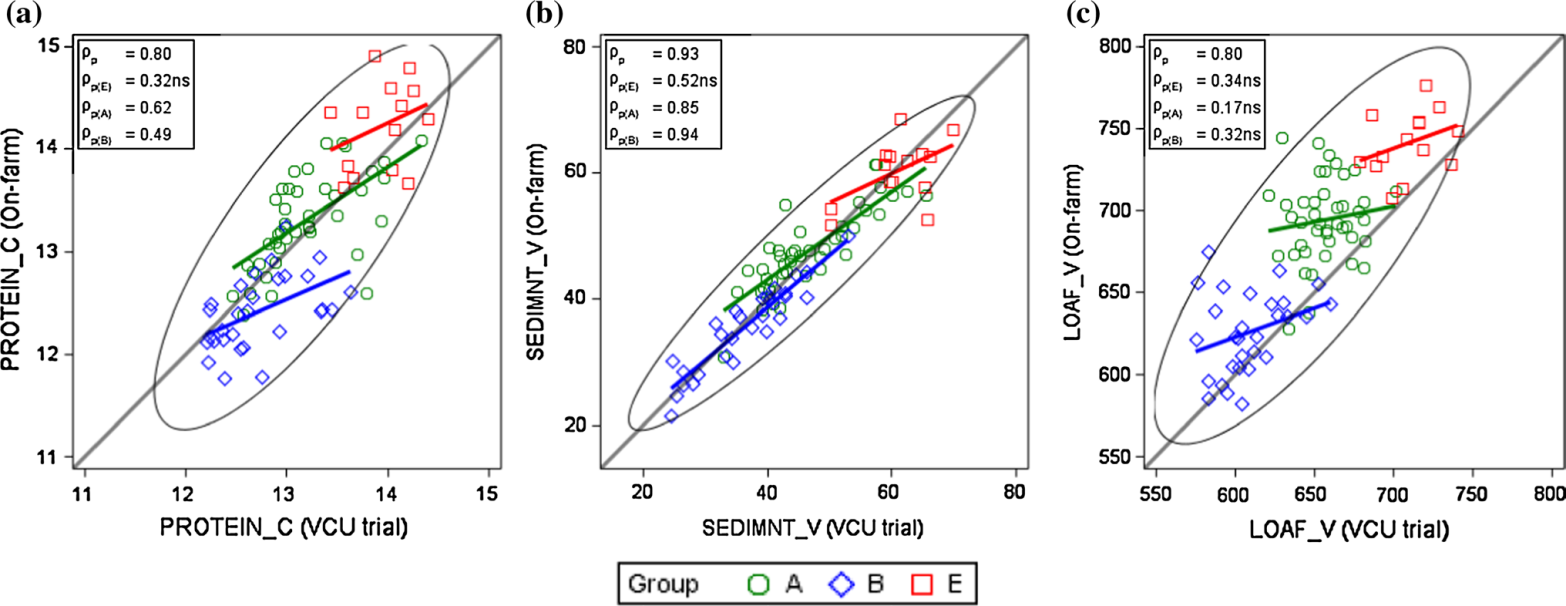
Correlation of adjusted variety means [*G*_*i*_ in Eq. (1)] for quality traits from VCU trials and on-farm (annual national survey) data including a 1:1 line. *ρ*_*p*_ phenotypic correlation coefficient over all varieties; *ρ*_*p*(.)_: phenotypic correlation coefficients within groups. ns: not significant different from zero at 1% level. *PROTEIN*_*C* crude protein concentration, *SEDIMNT*_*V* sedimentation value, LOAF_V loaf volume, on-farm loaf volume (calculated)

We further compared the adjusted variety means from VCU trial and from on-farm data, and plotted the correlation diagrams as shown in Fig. 8. There were 86 varieties in common. Figure 8a–c demonstrates that associations for protein concentration (*ρ*_p_ = 0.80) and loaf volume (*ρ*_p_ = 0.80) were strong, whereas correlation for sedimentation value was very strong, reaching *ρ*_p_ = 0.93. This result clearly shows that variety means for sedimentation values are much more alike in VCU trials and on-farm than for protein concentration and loaf volume. For all three traits, correlation coefficients within groups are much lower than the overall correlation, except for sedimentation value for groups A and B.

## Discussion

To give an overview to results from published studies for grain yield and quality, we summarized relevant parameters in Table 6.

**Table 6:** Summary of relevant studies on grain yield and quality of wheat

Author(s)	Region	Description	Relation of GRAIN_Y and PROTEIN_C^#^	Genetic trend		Phenotypic correlation					
				GRAIN_Y^a^	PROTEIN_C^b^	GRAIN_Y			PROTEIN_C		SEDIMNT_V
						PROTEIN_C	SEDIMNT_V	LOAF_V	SEDIMNT_V	LOAF_V	LOAF_V
This study (2016)	Germany	(a) VCU trials (b) on-farm (Tables 3, 5; Fig. 7)	(a) 8.3^***^ (b) $$\underline{{-0.071}^{***}}$$	(a) 0.559^***^	(a) −0.028^***^ (b) 0.016^*^	(a) −0.77^**^	(a) −0.42^**^	(a) −0.46^**^	(a) 0.67^**^ (b) 0.84^**^	(a) 0.67^**^ (b) 0.88^**^	(a) 0.77^**^ (b) 0.96^**^
Simmonds (1995)	Europe	Review of 13 published grain–protein relations	−7.1^n^								
Graybosch et al. (1996)	Nebraska, USA	30 G(varieties + lines), 2 Y(1990–1991),17E							0.73^**^	0.74^**^	
Smith and Gooding (1999)	England	36G, Y (1975–1995).220 GxY means from national wheat quality survey			−0.031^***^						
Oury and Godin (2007)	Northern+ southern France	21 bi-annual VCU trial series (1994–2005), 29–42 G per series, 458E	$\underline{{-0.082}^{\rm n}}$			−0.61^n^					
Kazman (2010)	Germany	459 G (first test year), 9 VCU trial series, 10 Y(1999–2008)				−0.72^n^	−0.41^n^	−0.51^n^	0.61^n^	0.62^n^	0.65^n^
Hartl et al. (2011), Mohler et al. (2011)	Germany	94 G (released 1961–2008, only dominating varieties with baking quality), 2Y, 5L, 7E,		0.245	0.041	−0.76^***^	−0.60^***^	−0.56^***^	0.85^***^	0.74^***^	0.81^***^
Oberforster and Werteker (2011)	Austria	VCU + regional trials, 31 Y(1980–2010)	Average of								
	(a) dry region	(a) (49H/18 N)G, 419E	(a) and (b):	(a) (0.35^n^/−)		(a) −0.48^**^/−0.73^***^	(a) 0.02/−	(a) 0.05/−			
	(b) humid region	(b) (13H/51 N)G, 514E	$\underline{{0.06}^{\rm n}}$	(b) (0.70^n^/−)		(b)−0.53^*^/−0.83^**^	(b) −/−0.04	(b) −/0.06			
Cormier et al. (2013)	France	225G (European high quality, released 1969–2010), 2Y (2009–2010), 3L, 4E		0.518^**^	−0.0457^***^						
Bilgin et al. (2015)	Northwest Turkey	36G (6 landraces, 24 varieties released 1960–2010, 6 lines), 2Y (2010–2011), 3L		0.32^**^	−0.022^*^						
Rozbicki et al. (2015)	Poland	7G, 3Y (2009–2010), 3L							0.77*	0.00	−0.03

*n* no significance test reported

*G* genotypes, *H* high quality (about E-grade), *N* normal bread quality (about A+B- grade), *Y* harvest years, *L* location, *E* environments

*GRAIN*_*Y* grain yield, *PROTEIN*_*C* crude protein concentration, *SEDIMNT*_*V* sedimentation value, *LOAF*_*V* loaf volume, on-farm loaf volume (calculated)

* Significant at 5% level

** Significant at 1% level

*** Significant at 0.1% level

# Regression slope of grain yield (dt ha^−1^) on protein concentration (%), underlined figure refers to regression slope of protein concentration on grain yield

^a^dt ha^−1^ year^−1^

^b^% year^−1^

### Yield and quality progress in VCU trials and on-farm

#### VCU trials

Our results showed a large significant genetic trend in grain yield, but simultaneously a significant negative genetic trend in protein concentration (Tables 3, 6). Most genetic trends reported in the literature are lower (Table 6), which can be ascribed to the fact that varieties with higher baking quality were grown, e.g. in the study of Hartl et al. (2011) (Table 6), whereas the decline for protein concentration was mostly stronger (Table 6).

Our results further showed that both traits are strongly negatively related, also within quality groups (Fig. 6a). If we expressed this relationship in terms of the regression of adjusted variety means for grain yield on protein concentration, we found a slope of −8.3 dt ha^−1^ (1% absolute change)^−1^, which says that an absolute increase of 1% in protein concentration resulted in a loss of 8.3 dt ha^−1^ grain yield (Table 6). The reciprocal relationship, i.e. regression of adjusted means for protein concentration on grain yield, indicated that a yield increase of 1 dt ha^−1^ causes an absolute loss of −0.071% protein concentration (Table 6). Similar results are found by Simmonds (1995), Oury and Godin (2007) and Oberforster and Werteker (2011) (Table 6). This negative relation between protein concentration and grain yield is genetically determined, as shown by several studies, e.g. Mohler et al. (2011) and Sherman et al. (2014). This makes it unlikely to simultaneously select genotypes with high yield and high protein concentration.

Despite the strong negative genetic relation between yield and protein concentration, our VCU results showed that wheat breeding in Germany was very successful in increasing yield by a rate which was about three times as high as the rate of decrease in protein concentration relative to 1983 (Table 3). Generally, our VCU trial results in Table 3 indicated that quality was partially improved. Specifically, we found a significant gain for hardness (13.4%) and milling yield (2.4%) relative to 1983. We further found a positive, yet not significant, gain for falling number (5.8%), sedimentation value (7.9%) and water absorption (1.2%) relative to 1983. Except for protein concentration, we found no significant negative genetic trends in the quality traits, which demonstrate that breeding against this major negative trend in protein concentration was effective. A surprise to us was the highly significant negative non-genetic trend for loaf volume, which indicated that non-genetic reasons are responsible for the loss of loaf volume. As this quality trait was tested in the same laboratory as on-farm samples, method of analysis should not be the reason for the contrasting results between trial and on-farm change in loaf volume. We were not able to find a plausible explanation for this observation.

Obviously, breeding efforts could not prevent a decline of protein concentration when raising yield level; however, breeding was successful in maintaining or moderately increasing protein quality. This becomes apparent by the observed improvement of sedimentation value in VCU trials (Fig. 2). This result is in accordance with the general knowledge that sedimentation value is a strong indicator for protein quality, and that protein quality is largely genetically determined, hence more variety specific (Payne et al. 1987; Graybosch et al. 1996; Wieser and Seilmeier 1998; Mohler et al. 2011).

Results from VCU trials have shown that elimination of C-graded varieties did not alter gain in grain yield very much, yet the positive impact on protein concentration, sedimentation value and loaf volume more than outweighed yield loss (Table 3). These results further corroborate the evidence that considerable progress was achieved in baking quality without appreciable losses in yield, when higher yielding C-group varieties are excluded. Regression lines in Fig. 4a–c demonstrate these results.

#### VCU trials vs on-farm

A very decisive point is the extent to which performance progress achieved in VCU trials transforms into on-farm progress. Comparison of progress of grain yield in VCU trials and on-farm indicates that enormous progress was achieved also on-farm. Moreover, significant genetic progress in baking quality as exemplified by the significant gain in sedimentation value and loaf volume apparently driven by the genetic component was achieved on-farm.

There are two main reasons why improvement in on-farm baking quality was higher than in VCU trials. First, one should be aware that varieties of both sources are grown under different agronomic but not under different environmental conditions. All VCU trial entries grown at the same location received year-specific identical treatment with respect to fertilizer and pesticide application, and crop management in order to ensure homogeneous testing conditions. Winter wheat varieties are graded into quality groups before they get released, which allows farmers to choose varieties with high quality grades. In contrast to VCU trials, on-farm grown varieties received variety-specific nitrogen fertilization, usually at higher rates and with late top dressing, and crop management according to their quality grade in order to obtain the best economic results for a varieties’ yield and baking quality. Second, the shift to varieties with higher baking quality was attractive to farmers due to large yield progress achieved in this segment. Loss in yield is compensated by higher prices when growing, e.g. a variety with A- instead of B-quality. Recently, released varieties with A-quality reached nearly the same yield level as varieties with B-quality (Fig. 4a).

As shown in Table 3, overall trend for grain yield in VCU trails was 0.77% p. a. relative to yield level 1983 (86.0 dt ha^−1^) and for on-farm 1.02% p. a. relative to yield level 1983 (60.9 dt ha^−1^). It is not surprising that on-farm yield is lower than for VCU trials for at least three reasons: Firstly, individual registration trials are dropped if they are not of sufficient quality in order to allow a fair comparison of entries, for example if frost damage, drought or lodging occurred. Secondly, the average age of a variety grown on-farm was about 10.5 years, whereas for trials it was only 3.5 years. This means that on-farm yields are lagging behind breeding progress observed in VCU trials by 7 years. Thirdly, perhaps most importantly are economic constraints such as grain prices and input (fertilizers, pesticides) costs (Fischer 2015).

Contrary to trial results, on-farm protein concentration slightly increased from an absolute level of 12.9–13.1% (1.5% relative to 1983) during 1983–2014 (Table 3) which may be mainly attributed to an increased growing of A-grade varieties with higher protein concentration. Also, a higher average N-fertilization could be involved, but we are not aware of any studies quantifying long-term changes of nitrogen application of wheat in Germany. Smith and Gooding (1999) observed from a UK quality survey between 1975 and 1995 that an increase of N-fertilization of 100 kg N ha^−1^ leads to an absolute gain in protein concentration of 1%. Cormier et al. (2013) and Wieser and Seilmeier (1998) provided further evidence of the effect of nitrogen supply on protein concentration and quality. Moreover, we found for on-farm data a significant gain for sedimentation value (45.4% relative to 1983) and loaf volume (8.3% relative to 1983) as compared to VCU trials (C-grade varieties excluded) where the relative gain for sedimentation value was 9.3% and the loss for loaf volume −7.0%. This difference may be attributed mainly to the absence of protein decrease on-farm (Table 3; Fig. 3).

### Genotype, environment and genotype by environment interaction in VCU trials

Our results are in agreement with general conclusions by other authors: (1) genotype and environment had an effect on quality parameters, (2) the contribution of genotype by environment interaction was considerably less than either environment or genotype (e.g. Finlay et al. 2007; Dencic et al. 2011), (3) yield and protein concentration was the most sensitive variable to environmental fluctuations (e.g. Hristov et al. 2010; Bilgin et al. 2015), and (4) parameters related to protein quality, reflected in glutenin concentration, were most genotype dependent (e.g. Graybosch et al. 1996; Bilgin et al. 2015).

Among all traits, except grain yield, variation of protein concentration was most highly influenced by location (15%) and location by year (45%) effects. Variation from year to year (5%) was of minor importance. This strong influence of location, as compared to year, is in agreement with results from Rozbicki et al. (2015). The remarkable effect of locations can be explained by a high influence of local growing conditions, especially nitrogen supply from soil as the principal factor affecting environmental variation in protein concentration and composition (Cormier et al. 2013).

Contrary to what we found for protein concentration, falling number as an indicator of starch quality fluctuation from year to year (20%) is about five times as large as for location (4%) (Fig. 5). This can be explained by the more year-related influences of temperature and rainfall during harvest time, which determines alpha-amylase activity in grain starch.

Among all traits, variation of sedimentation value was most strongly influenced by genotypes (69%) clearly confirming that protein quality is genetically determined to a very high degree (e.g. Payne et al. 1987). This may be explained by the sedimentation value use in the early breading process to select genotypes for high baking quality as reported, e.g. by Knott et al. (2009), Hartl et al. (2011) and Souza et al. (2012).

### Phenotypic and genotypic correlation of quality traits in VCU trials and on-farm

#### VCU trials

Results, as presented in the correlation matrix of Table 5, indicate differences between genotypic and phenotypic values, which are largest for grain yield and smaller for protein concentration and falling number. The differences between both measures of correlation may be explained by the large variation due to environment and genotype by environment interaction as compared with genotypic variation for the aforementioned traits. For traits with low genotypic variances, the genotypic effects in the estimated variety means are masked by environmental variation to a greater extent than is the case for means from traits with higher genotypic variation. Consequently, the phenotypic correlation may underestimate the genetic relation between traits. Comparison of phenotypic and genetic correlation of grain yield with sedimentation value (*ρ*_p_ = −0.42, *ρ*_g_ = −0.73) and sedimentation value with loaf volume (*ρ*_p_ = 0.77, *ρ*_g_ = 0.79) demonstrates this difference (Table 5).

Our long-term results from correlation analysis confirmed the well-known strong negative relationship between protein concentration and yield and its positive relationship with baking quality traits. The negative relation between grain yield and protein concentration also holds for correlation within quality groups as shown in Fig. 6a, indicating that this relation generally cannot be broken easily because of its partially genetic basis (Mohler et al. 2011; Sherman et al. 2014; Kaya and Akcura 2014). The phenotypic correlation coefficient for grain yield–protein relation in this study is of about of the same magnitude as results from other studies shown in Table 6, except for the set of “high quality” varieties from Austrian trials which have lower coefficients (Table 6). In the Austrian study, “high quality’’ varieties correspond to E-grade quality which showed similar values (Fig. 6a). Good agreement of correlation coefficients from our study with results from other studies shown in Table 6 were found for grain yield with sedimentation value and loaf volume, and between other quality traits, except for some results from the Austrian and Polish studies.

#### On-farm

On-farm phenotypic correlations of protein concentration, sedimentation value and expected loaf volume are considerably stronger than in VCU trials (Figs. 6, 7). This can partially be attributed to the fact that for each variety there were results from 6 years available on-farm and only about 3.5 years in trials on the average. A further reason may be that expected loaf volume was determined by a functional relationship with sedimentation value and protein concentration leading to a stronger correlation.

#### VCU trials and on-farm

Treatment of VCU trials and on-farm crop management and also growing years were quite different. Despite differences in trial and on-farm crop management, we found a good agreement of adjusted variety means from VCU trials and on-farm data for protein concentration, sedimentation value and loaf volume, which points to the variety specific nature of these traits (Fig. 8). Especially the very strong correlation of sedimentation value (*ρ*_p_ = 0.93) demonstrates that protein quality is to a high degree variety specific and genetically determined.

## Conclusions

In VCU trials, large progress has been made in raising grain yield during the last 32 years. But the well-known strong negative and genetically controlled relationship with protein concentration leads to a considerable loss in protein concentration. On the other hand, protein concentration is closely associated with key traits for baking quality, i.e. sedimentation value, and loaf volume. Those unfavourable relations provide a great challenge for wheat breeding aimed at raising grain yield, and simultaneously maintaining or increasing level of baking quality. When taking into account the large gain in grain yield and the negative relationship with protein concentration, our results indicated that losses in baking quality were mitigated by improved protein quality. The apparent gain of the highly genetically determined trait sedimentation value provided evidence that progress in baking quality was achieved mainly due to improved protein quality.

Grain yield and protein concentration are highly influenced by environmental factors, whereas variation in sedimentation value, hardness, water absorption and loaf volume is predominantly governed by the genotype.

On-farm grain yield gained at the same magnitude as VCU trial yield in terms of absolute values, however, at a lower level. On-farm progress in quality traits clearly exceeds that observed in VCU trials; for protein concentration even a positive trend was observed on-farm. For all on-farm traits, genetic trends were significant and dominating. It is not surprising that baking quality has been more improved on-farm than in VCU trials, because farmers shifted continuously to varieties with better baking quality and were able to apply optimal variety-specific crop management. In VCU trials, however, varieties in each quality group did not change over the 32 years.

Our study demonstrated that for VCU trials, strong to very strong relations exist among protein concentration, sedimentation value and loaf volume, and that this relation was even stronger for on-farm data. Adjusted variety means from VCU trial and on-farm data are strongly related for protein concentration and loaf volume, and very strong for sedimentation value which again confirms the highly variety-specific and genetically controlled nature of this trait.

### Author contribution statement

FL conceived the study, carried out the analyses, prepared the figures and tables and wrote the manuscript. HPP provided advice on statistical analysis, DR in using and interpreting data for baking quality. Both read and amended the paper. TD and UM assembled all datasets, prepared and formatted them for statistical analysis. Both participated in editing the paper. AH was responsible for carrying out laboratory tests for VCU trial and national harvest survey samples.

## Electronic supplementary material

Below is the link to the electronic supplementary material.

Supplementary material 1 (DOCX 31 kb)

Supplementary material 2 (DOCX 81 kb)
