# Supplementary material for: Breeding progress, environmental variation and correlation of winter wheat yield and quality traits in German official variety trials and on-farm during 1983–2014
Source: Theor Appl Genet. 2016 Oct 27;130(1):223–45. doi: 10.1007/s00122-016-2810-3 (PMC5215243; doi:10.1007/s00122-016-2810-3)
Supplement: Supplementary file 1 — Supplementary material 1 (DOCX 31 kb) [file 122_2016_2810_MOESM1_ESM.docx]

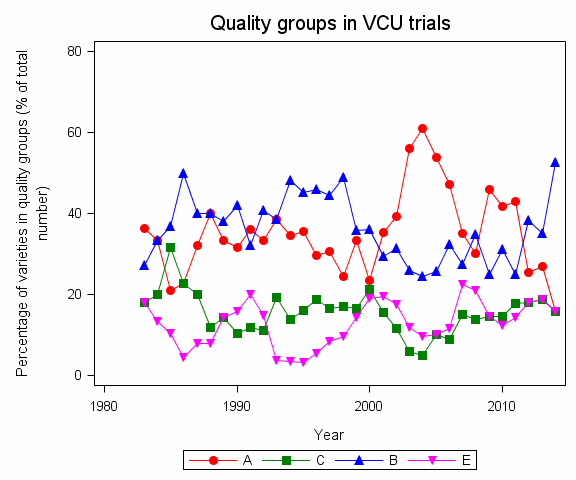


**Electronic Appendix Fig. S1**: Number of varieties in quality groups as percentage of total number of varieties within a year.
