# Supplementary material for: Breeding progress, environmental variation and correlation of winter wheat yield and quality traits in German official variety trials and on-farm during 1983–2014
Source: Theor Appl Genet. 2016 Oct 27;130(1):223–45. doi: 10.1007/s00122-016-2810-3 (PMC5215243; doi:10.1007/s00122-016-2810-3)
Supplement: Supplementary file 2 — Supplementary material 2 (DOCX 81 kb) [file 122_2016_2810_MOESM2_ESM.docx]

a) b)


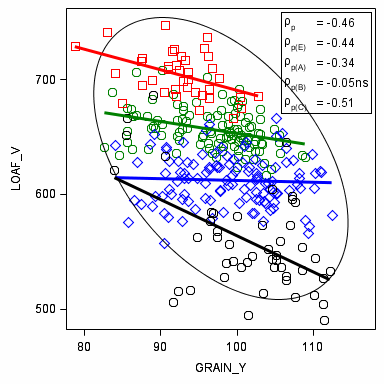

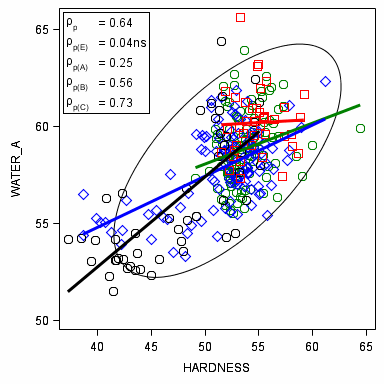


c) d)


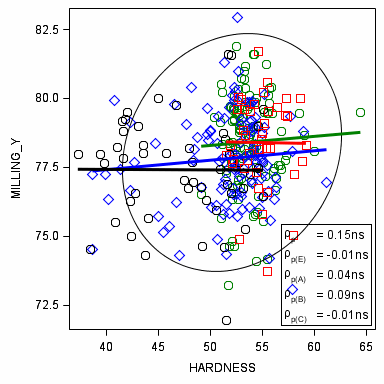

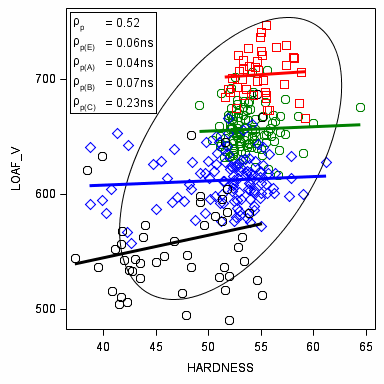


**Electronic Appendix Fig. S2**: Phenotypic correlation of adjusted variety means [*G_i_* in Eq. (1)] for quality traits from VCU trials. Quality groups with grades in descending order are E: elite wheat, A: quality wheat, B: bread wheat, C: others.

ρ_p_ : phenotypic correlation coefficient over all varieties; ρ_p(.)_: phenotypic correlation coefficients within groups.

ns: not significant different from zero at 1% level

GRAIN_Y grain yield; HARDNESS hardness; WATER_A water absorption; MILLING_Y milling yield; LOAF_V loaf volume.
